# COVID‐19 and myasthenia gravis: A review of neurological implications of the SARS‐COV‐2

**DOI:** 10.1002/brb3.2789

**Published:** 2022-10-28

**Authors:** Syed Muhammad Ismail Shah, Farah Yasmin, Roha Saeed Memon, Nadia Nazir Jatoi, Ilma Saleh Savul, Sana Kazmi, Syed Abdullah Monawwer, Muhammad Daim Bin Zafar, Muhammad Sohaib Asghar, Muhammad Junaid Tahir, Ka Yiu Lee

**Affiliations:** ^1^ Department of Internal Medicine Ziauddin Medical University Karachi Pakistan; ^2^ Department of Internal Medicine, Dow Medical College Dow University of Health Sciences Karachi Pakistan; ^3^ Department of Internal Medicine St. Joseph Medical Center Houston Texas USA; ^4^ Department of Internal Medicine Dow University Ojha Hospital Karachi Pakistan; ^5^ Lahore General Hospital Lahore Pakistan; ^6^ Swedish Winter Sports Research Centre, Department of Health Sciences Mid Sweden University Östersund Sweden

**Keywords:** Guillain–Barre syndrome, immunoglobulins, infection, myasthenia gravis, pyridostigmine bromide, severe acute respiratory syndrome coronavirus 2

## Abstract

**Introduction:**

This review highlights the potential mechanisms of neuromuscular manifestation of COVID‐19, especially myasthenia gravis (MG).

**Methods:**

An extensive literature search was conducted by two independent investigators using PubMed/MEDLINE and Google Scholar from its inception to December 2020.

**Results:**

Exacerbations of clinical symptoms in patients of MG who were treated with some commonly used COVID‐19 drugs has been reported, with updated recommendations of management of symptoms of neuromuscular disorders. Severe acute respiratory syndrome coronavirus 2 can induce the immune response to trigger autoimmune neurological disorders.

**Conclusions:**

Further clinical studies are warranted to indicate and rather confirm if MG in the setting of COVID‐19 can pre‐existent subclinically or develop as a new‐onset disease.

## 1 INTRODUCTION

The severe acute respiratory syndrome coronavirus 2 (SARS‐CoV‐2) was first initiated as unexplained bilateral pneumonia and acute respiratory distress occurring in the city of Wuhan (Singhal, 2020). The SARS‐CoV‐2 is also known to be neuro‐virulent. Previously, the spread of the SARS‐CoV virus through first‐ or second‐order connections from the olfactory nerve to regions of the cortex, brainstem, and midbrain as well as poorly connected regions such as the thalamus and brainstem have been demonstrated on transgenic mice (Netland et al., 2008). Another possible route is through retrograde neuronal spread from the lungs through the vagus nerve (De Virgiliis & Di Giovanni, 2020). Transsynaptic transfer starting from the peripheral nerves to the central nervous system (CNS) has been seen in other beta coronaviruses as well (Li et al., 2020).

In general, neurological symptoms comprise one of the clusters of COVID‐19 manifestations. Olfactory and gustatory dysfunctions are common presenting symptoms of mild‐to‐moderate COVID‐19 infection (Lechien et al., 2020), whereas cerebrovascular accident and altered mental status following encephalopathy or that meeting the criteria for a neuropsychiatric diagnosis like psychosis, dementia‐like neurocognitive syndrome, and affective disorders have also contributed to the cluster (Varatharaj et al., 2020). This review aims to highlight the different neurological complications of COVID‐19 and the difficulties arising in their treatment, especially in neuromuscular disorders such as myasthenia gravis (MG).

## 2 METHODS

An extensive literature search was conducted by two independent investigators using PubMed/MEDLINE and Google Scholar from its inception to December 2020. The following search string was employed: “SARS‐CoV‐2” AND “central nervous system” OR “peripheral nervous system” OR “MG” OR “myasthenia crisis” OR “diagnosis” OR “treatment” OR “recommendations”. We excluded all articles for which full texts were not available, and those in a language other than English.

## 3 RESULTS

### 3.1 AChR antibody‐positive myasthenia gravis development in association with COVID‐19

Myasthenia gravis is an autoimmune neuromuscular disease that results from the antibodies that block or destroy the nicotinic acetylcholine (Ach) receptors or their components in the postsynaptic membrane at the neuromuscular junction (Phillips & Vincent, 2016). This hinders the neuromuscular transmission of nerve impulses impairing muscle contractions, ultimately leading to muscle weakness and fatigue. In contrast to other neurological and psychological manifestations, the association of myasthenia gravis with COVID‐19 has only been well described in the context of risk factors for severe COVID‐19 infection at presentation (Camelo‐Filho et al., 2020). That is, patients with a pre‐existing diagnosis of myasthenia gravis when hospitalized for superimposed COVID‐19 infection, had severe courses of the COVID‐19 disease (Camelo‐Filho et al., 2020). However, only recently, reports of positive COVID‐19 patients with subsequent development of symptoms of myasthenia gravis have surfaced (Restivo et al., 2020). A study conducted by Restivo et al. (2020) described three cases belonging to the high‐risk elderly group. This time frame from the onset of COVID‐19 symptoms to that of myasthenia gravis symptoms is consistent with the case of other neurological disorders, such as Guillain–Barre syndrome (GBS; Toscano et al., 2020). It was the first report cases that developed myasthenia gravis following COVID‐19 infection (George, 2020). MG post viral infections have been previously reported with Epstein Virus, post varicella, and pharyngitis infections (Tahiri A et al. 2022). More recently, a systematic review (Tugasworo et al., 2022) showed that there were almost six studies about new‐onset of MG after COVID‐19 infection. The plausible reasons for this have been attributed to the structural similarities between Ach receptor and SARS‐CoV‐2 receptor, latent autoimmune infection's reactivation, and hyper immune response like multisystem inflammatory syndrome in children may be the possible explanation of it. Another case report from Northern Iran also showed development of new‐onset MG following COVID‐19. The findings of these studies highlight the role of autoimmunity in neurological disorders associated with COVID‐19. It also allows doctors to keep an eye out for a possible parainfectious profile of myasthenia gravis and other autoimmune neurological disorders in COVID‐19 positive cases, similar to that seen in the case of superimposed GBS in COVID‐19 cases (Zhao et al., 2020).There has been a long understanding of the fact that AChR antibody‐positive myasthenia gravis is usually unmasked in the setting of severe viral and bacterial infections. This can be supported by the fact that AChR antibodies are the kind that gradually peaks up to levels as high as those reported in the study by Restivo et al. (2020). This makes the occurrence of newly onset myasthenia gravis 5–7 days after COVID‐19 positivity unlikely rather, a more logical explanation could be that these patients had pre‐existing subclinical myasthenia gravis. As these patients got infected with COVID‐19, the widespread immunological response to the SARS‐CoV‐2 virus also led to ramped‐up antibodies against AChR resulting in a more clinically apparent myasthenia gravis (Brooks, 2020).

In addition to this, about 8% of myasthenia cases have muscle‐specific kinase (MuSK) antibodies (Evoli et al., 2018). The mechanisms underlying AChR antibody myasthenia gravis (AChR‐MG) are different from those of MuSK antibody myasthenia gravis (MuSK‐MG). Couple of studies have also reported the association between MuSK antibody‐associated myasthenia gravis and SARS‐CoV‐2 (Assini et al., 2021; Muhammed et al., 2021). The underlying mechanism of MuSK‐associated Myasthenia Gravis is more likely the breakdown in self‐tolerance mechanisms than cross‐reactivation given their molecular differences.

### 3.2 Myasthenia gravis and vaccinations

New MG case reports following COVID‐19 vaccination have also emerged (Lee et al., 2022). One case report documents a 33‐year‐old female who developed rapid MG following the second dose of a Pfizer‐BioNTech COVID‐19 vaccination. Previously, a male patient with late onset of MG was described after receiving the first dose of COVID‐19 vaccination (Chavez & Pougnier, 2021). Another case report by Kang et al. (2022) also reported a 35‐year‐old male patient developing MG with ocular symptoms following the second dose of ChAdOx1 nCoV‐19 vaccination. Similarly, another case reported of ocular MG induced by the viral vector Oxford‐AstraZeneca coronavirus disease (COVID‐19) vaccine (Maher et al., 2022).Vaccines, particularly that with bovine, yeast, and chick antigens can induce subclinical autoimmunity as they synthesize antibodies that have the potential to cross‐react with AChR or MuSK protein, both causing myasthenia gravis (Arumugham, 2019).Infection, in this case with the SARS‐CoV‐2 virus, can then lead to a recall response of vaccine‐induced autoimmunity mediated by memory cells (Restivo et al., 2020).

In conclusion, further studies are needed to confirm the mechanisms of development of myasthenia gravis symptoms after COVID‐19 infection positivity. First, these studies should be able to indicate and confirm if myasthenia gravis was pre‐existent subclinically or new‐onset. If the latter is confirmed, additional research could be employed to discuss the particular role of SARS‐CoV‐2‐induced immune response in triggering autoimmune neurological disorders, myasthenia gravis in particular. However, if the pre‐existent disease is well established, these results could be utilized to analyze potential factors eliciting subclinical autoimmunity

In addition to new‐onset MG following vaccinations, the safety of vaccinations in patients with pre‐existing MG was investigated and a study reported that the inactivated COVID‐19 vaccines might be harmless in patients with MG with Myasthenia Gravis Foundation of America (MGFA) score classification I to II, demonstrating the recommendation to promote vaccination for MG patients during the still expanding COVID‐19 pandemic (Ruan et al., 2021). Another study demonstrated particularly better short‐term safety in MG patients, who can take advantage of vaccination to avoid life‐threatening severe complications such as COVID‐19 pneumonia (Lupica et al., 2022). Another cohort study reported the safety and tolerability of mRNA COVID‐19 vaccines in MG patients, particularly for those patients who could be at higher risk of complications if exposed to SARS‐CoV‐2 infection (Farina et al., 2022). One more study provides Class IV evidence that patients with MG receiving the mRNA‐1273 vaccine did not show clinical worsening after vaccination and that most of the patients achieved high cellular or immune response levels. From aforementioned studies, it is demonstrated that COVID‐19 vaccinations were relatively well tolerated in patients with pre‐existing MG and were not associated with worsening severity of their MG, instead can potentially prevent life‐threatening complications by COVID‐19 in these patients.

### 3.3 Myasthenia crisis due to COVID‐19

Patients with pre‐existing myasthenia gravis, either subclinical or clinically apparent, if infected with COVID‐19 can present with myasthenia crisis (Delly et al., 2020). Myasthenia gravis characterized by severe muscle weakness may compromise respiratory function that requires intubation and mechanical ventilation (Wendell & Levine, 2011). In general, infections have been concluded as the most common cause of MG exacerbation in one retrospective study (Gummi et al., 2019). This exacerbation can not only unmask subclinical myasthenia gravis but also worsen both subclinical and clinical myasthenia gravis leading each to the crisis (Kusnar & Kaminski, 2015). Consistently, increased rates of worsening of the disease in those with clinical myasthenia gravis and increased incidence of new presentations in cases with undiagnosed subclinical myasthenia gravis could be expected during the COVID‐19 pandemic (Guidon & Amato, 2020).

The first known case of concomitant presentation of myasthenia crisis and COVID‐19 was reported by Delly et al. (2020). The case involved a 56‐year‐old woman with a known history of myasthenia gravis for over 5 years well managed on pyridostigmine, prednisone, and intravenous immunoglobulin (IVIG). The patient was also taking hydroxychloroquine for mixed connective tissue disease. Her disease exacerbations in the past had only included lower extremity weakness, ptosis, dysphagia albeit, but no history of mechanical ventilation was reported. The initial chest x‐ray was consistent with pneumonia and the patient was started on antibiotics. However, on testing positive for COVID‐19, antibiotics were discontinued and the patient was put back on hydroxychloroquine. Neurological examination revealed motor strength of 4/5 in proximal upper and lower extremities, whereas speech and swallowing could not be assessed due to intubation. However, considering the poor respiratory function and former positive history of myasthenia gravis, the patient was restarted on pyridostigmine and IVIG. The patient was extubated after five IVIG doses owing to improvement on ventilator settings. However, the motor strength in the proximal extremities worsened (2/5) that required the patient another IVIG course for 2 days. This led to an improvement in the physical activity of the patient by day 25 of admission (Delly et al., 2020).

This case, similar to other relevant literature, does not document COVID‐19 as the cause of the development of the myasthenia crisis (Delly et al., 2020). Similarly, interrelations of symptoms, clinical outcomes, and treatment regimens in pregnant women, diagnosed with myasthenia gravis and superimposed COVID‐19 infection was also investigated. But, it yielded that the co‐occurrence of MG and COVID‐19 infection in pregnancy does not elicit exacerbation in either of those conditions (Neykova et al., 2021). However, since infections, in general, have been known to trigger and augment autoimmunity (Gilhus et al., 2018), this case can be taken as an initial step to establish the causal relationship between COVID‐19 and myasthenia crisis. In one study, a third of the Australian patients revealed infections as the cause of their myasthenia gravis exacerbation, whereas around half of this population claimed seasonal variation to play a role in the worsening of their disease (Blum et al., 2015). Consistently, another study on a large US cohort also confirmed seasonal variation as the likely underlying cause of myasthenia gravis exacerbation (Melamed et al., 2014). Both studies concluded that infection, respiratory and urinary tract infection in particular, is the most probable cause of the seasonal pattern of myasthenia gravis exacerbation. Another Spanish population‐based study concluded infection as the most common trigger that precipitates a life‐threatening event such as respiratory and swallowing dysfunction in known cases of myasthenia gravis (Ramos‐Fransi et al., 2015). Though discontinuation of treatment was reportedly the most common cause, the infection was the second most common factor that relapsed myasthenia gravis in a Chinese cohort of patients with childhood onset of disease (Gui et al., 2015).

Viruses can trigger and augment autoimmunity through molecular mimicry, epitope spreading, enhance T‐cell signaling, and upregulation of a series of cytokines and co‐stimulatory molecules (Gilhus et al., 2018). Molecular mimicry, epitope spreading, and additional effects of pathogens are suspected to play a role in the initial development of autoimmune diseases, whereas bystander activation due to the underlying inflammatory environment of infections and/or superantigens might accelerate autoimmune responses later on in the disease course (Münz et al., 2009). Considering SARS‐CoV‐2 in particular, a shared component between myasthenia crisis and COVID‐19 infection pathophysiology is cytokine dysregulation. The development of a cytokine storm primarily due to COVID‐19 infection could be the mechanism that also accelerates pre‐existing auto‐reactive immune responses against self‐antigens. This common underlying immunopathogenesis could be considered a plausible explanation for the co‐existence of COVID‐19 infection and myasthenia crisis in the case discussed herein (Delly et al., 2020).

### 3.4 Review of treatments prescribed for COVID‐19 infection in myasthenia gravis patients

Treatments aimed at alleviating COVID‐19 infection in myasthenia gravis patients are reviewed. One case reported the use of a combination of hydroxychloroquine, favipiravir, oseltamivir, along with meropenem and subcutaneous low‐molecular‐weight heparin (LMWH). The patient was also being administered pyridostigmine, which she had been previously maintained on for 4 years (Aksoy & Oztutgan, 2020). Hydroxychloroquine regulates pro‐inflammatory cytokines such as IL‐1, IL‐6, and tumor necrosis factor‐α and inhibits viral entry. In addition, it affects lysosomal activity and can change the intracellular pH. However, hydroxychloroquine was terminated in this case after neurological evaluation, followed by administration of linezolid and methylprednisolone. Broad‐spectrum antibiotics such as carbapenems and linezolid prevent the likelihood of secondary infections in COVID‐19 patients (Clancy et al., 2020). Hydroxychloroquine is not necessarily an effective treatment choice for improving clinical symptoms as demonstrated by the Outcomes Related to COVID‐19 Treated with Hydroxychloroquine among Inpatients with Symptomatic Disease (ORCHID) trial (NIH, 2020).

#### 3.4.1 Anticoagulation

A hypercoagulable state in severe COVID‐19 cases can increase the risk of thrombosis. There is also an established association of elevated D‐dimer levels and venous thromboembolism with a worse prognosis in patients. Therefore, anticoagulation has been recommended as a preventive therapy for thrombosis in COVID‐19 treatment. In particular, LMWH has been postulated to have other non‐anticoagulant benefits for COVID‐19 (Buijsers et al., 2020). The respiratory system has a protective endothelial barrier in the form of the glycocalyx whose main contributor to the glycocalyx barrier is a sulfated glycosaminoglycan called heparan sulfate (HS). Heparinase (HPSE) activity can disrupt this barrier function, causing severe clinical outcomes in COVID‐19. LMWH can be used to inhibit the activity of HPSE, therefore being protective of the glycocalyx barrier function (Buijsers et al., 2020).

#### 3.4.2 Antivirals

Favipiravir is an antiviral drug that has been previously used against influenza and Ebola infections (Agrawal et al., 2020). It undergoes phosphoribosylation to favipiravir‐RTP and inhibits the activity of the RNA‐dependent RNA‐polymerase (RdRp) enzyme. An oral dose of 1800 mg on day 1 followed by 800 mg twice daily on subsequent days was used in a Japanese observational study group, showing some promising results in mild and moderate disease. At day 7, 73.8% and 66.6% of patients with the mild and moderate disease showed clinical improvement respectively, while only 40.1% of patients with the severe disease showed clinical improvement (Agrawal et al., 2020). Similarly, a prospective, open‐label multicenter trial in China compared two treatment arms in moderate COVID‐19, one with favipiravir at a dose of 1600 mg twice daily followed by 600 mg twice daily, and the other with conventional therapy and 200 mg of umifenovir thrice a day. On day 7, clinical improvement was seen in 71.43% of favipiravir‐treated patients compared to 55.86% of the umifenovir‐treated patients (Agrawal et al., 2020).

Oseltamivir is also an antiviral drug, reserved for the treatment of influenza. It is a neuraminidase inhibitor that has mostly been used in comparison groups in clinical trials investigating COVID‐19 treatments (Sanders et al., 2020). Other antivirals include lopinavir and remdesivir. The replication and survival of the SARS‐CoV‐2 can depend on the cleavage of polyproteins by 3‐chymotrypsin‐like protease and papain‐like protease, similar to other viruses of the Orthocoronavirinae family (Hossen et al., 2020). Lopinavir targets these protease enzymes, and ritonavir inhibits the enzyme cytochrome P450, increasing the plasma concentration of lopinavir (Scavone et al., 2020). Therefore, strict monitoring is needed due to potential drug–drug interactions. Cao et al. enrolled 199 patients, where 99 of them received combination treatment with lopinavir/ritonavir and standard of care while the remaining 100 only received standard of care treatment. The results of the study showed no clinical improvement in the combination group. In fact, there were more gastrointestinal side effects seen in the combination group. Remdesivir inhibits viral replication by acting on RdRp. The Adaptive COVID‐19 Treatment Trial (ACTT‐1) investigated its role in COVID‐19 patients where they received an intravenous infusion of remdesivir or placebo for up to 10 days. Remdesivir‐treated patients showed decreased recovery time and decreased patient mortality compared to the placebo group (Beigel et al., 2020).

#### 3.4.3 Immune‐modulating drugs

In addition to administration of IVIG and pyridostigmine, there have been some cases where immunosuppressants such as corticosteroids have been used in myasthenia gravis patients show good results (Rein et al., 2020). Corticosteroids act on the glucocorticoid receptor and generally have two mechanisms. They inhibit genes that are involved in regulating the expression of cytokines such as IL‐4, IL‐10, IL‐13, and TGFβ, and promote transcription of anti‐inflammatory genes (Burrage et al., 2020). Treatment with corticosteroids for SARS‐CoV‐2 is controversial in terms of previous experiences with SARS infection. In one study patients experienced adverse outcomes despite being younger and having fewer comorbidities (Auyeung et al., 2005). The recovery trial brought more clarity to the issue when there was a 35% reduction in mortality among patients on invasive mechanical ventilation. Therefore, corticosteroids are more beneficial to critically ill patients or may be useful in preventing an extended cytokine storm. However, they can potentially cause immunosuppression, increase viral replication, and may not be recommended for routine care (Rizk et al., 2020).

Tocilizumab has also been administered in a case report of two females with refractory myasthenia gravis, where they had failed to respond to rituximab (Jonsson et al., 2017). Tocilizumab binds to membrane‐bound IL‐6 receptor and soluble IL‐6 receptor, which eventually blocks the binding of IL‐6, inhibiting signal transduction (Copaescu et al., 2020). Therefore, it is used to prevent cytokine storms in COVID‐19 patients. In two studies investigating its effect on COVID‐19 patients, it was capable of reducing the acute phase reactants. One study enrolled 15 patients, where 8 of them received a combination treatment of methylprednisolone and tocilizumab. Tocilizumab administration reduced the elevated C‐reactive protein (CRP) levels in the patients (Luo et al., 2020). The second study included 21 patients and saw favorable outcomes in the majority of the patients with a decrease in CRP, an improvement in symptoms and CT opacity changes, and a decrease in the lymphocyte percentage (Xu et al., 2020).

### 3.5 Neuromuscular symptoms exacerbated by common treatments prescribed for COVID‐19

Hydroxychloroquine has been documented to induce new‐onset myasthenia gravis as a young patient taking the drug to manage her systemic lupus erythematosus presented with muscle weakness (Varan et al., 2015). Similarly, in another study, patients on treatment with hydroxychloroquine developed new‐onset myasthenia gravis (Jallouli et al., 2012). However, one out of a few with established myasthenia gravis diagnosis well maintained on therapy, developed acute disease exacerbation when administered with hydroxychloroquine for 3 weeks (Jallouli et al., 2012). However, as the symptoms continued to persist despite discontinuation of treatment in both patient groups, it could be suspected that the symptoms of myasthenia occurred by chance and not due to hydroxychloroquine (Jallouli et al., 2012). In contrast, the association of chloroquine and myasthenia gravis is well established as new‐onset myasthenia symptoms have also been reported with the use of chloroquine (Jallouli et al., 2012). In cases with AChR antibody‐positive myasthenia gravis, antibodies returned to undetectable levels as soon as the treatment was withdrawn (Jallouli et al., 2012). These findings establish the role of chloroquine in reversible induction of the immune system with resultant anti‐AChR antibody production (Jallouli et al., 2012). However, in cases negative for anti‐AChR antibodies, a direct toxic effect of chloroquine on the neuromuscular junction can be argued (Jallouli et al., 2012). Two different potential mechanisms, therefore, may be involved.

Another treatment for COVID‐19 management alongside chloroquine and hydroxychloroquine is azithromycin from the macrolide group (Guidon & Amato, 2020). However, azithromycin has also been reported to worsen myasthenia gravis (Guidon & Amato, 2020). In one study that documented the medications that could aggravate myasthenia gravis and is generally avoided or used with caution, azithromycin topped the list (Gummi et al., 2019). Though prescription of azithromycin or other high‐risk medications was mostly associated with an exacerbation within the initial days of drug use, flare‐ups continued to be reported up to 30‐days period following initiation of medication. These medications proved only second to infections as significant predictors of myasthenia‐related emergency department visits and hospitalizations (Gummi et al., 2019). A study found improvement in azithromycin‐induced myasthenia crisis with IV calcium gluconate and suggested a probable mechanism for this disease exacerbation (Pradhan et al., 2009). Since calcium gluconate is known to potentiate the presynaptic release of Ach, the relief in symptoms could mean that azithromycin induced a block in Ach release at the presynaptic neuromuscular junction (Pradhan et al., 2009). However, definite pathophysiological conclusions based on myasthenia gravis exacerbation with azithromycin and subsequent improvement with IV calcium gluconate cannot be drawn from the case study of a single patient. Nonetheless, considering the potential risk of disease aggravation, azithromycin has been contraindicated in myasthenia gravis patients including those with congenital variants of the disease (Solé et al., 2020). However, it was also recently reported that in patients with prior MG, ongoing long‐term immunosuppressive immunotherapy to MG should be maintained during the COVID‐19 pandemic and that Azithromycin (AZM) can be used safely in MG patients and concurrent COVID‐19 infection (Zakaria Saied 2021).

The first study to report the co‐existence of myasthenia crisis and COVID‐19 also postulates the potential role of experimental COVID‐19 medications in the worsening of myasthenia gravis in addition to the risk associated with the COVID‐19 infection itself (Delly et al., 2020). The patient was administered a combination of hydroxychloroquine and azithromycin to manage COVID‐19. Considering that the patient needed additional doses of IVIG to regain motor strength, it could be hypothesized that these medications had added to the exacerbation caused by the infection alone (Delly et al., 2020). In conclusion, these drugs should be used with caution, if unavoidable in the myasthenia gravis patient cohort. Particularly in those already intubated and ventilated, these drugs can be considered in the absence of alternative treatments albeit, in the rest, these are generally not recommended but rather contraindicated (Solé et al., 2020).

### 3.6 Recommendation for emergency management/treatment of neuromuscular symptoms during COVID‐19

Currently recommendations on how to treat myasthenia gravis in COVID‐19 patients vary. According to a joint consensus by an myasthenia gravis expert panel, patients with pre‐existing myasthenia gravis should continue to take their respective medications unless advised by their physician since these drugs have longer‐lasting effects, are washed out late, and the rebuilding of effects can take a while (Jacob et al., 2020). This can be demonstrated by a case series of four patients with generalized myasthenia gravis and COVID‐19 which concluded that existing immunosuppressant medications are not advised to be halted (Hübers et al., 2020). Furthermore, according to the consensus, patients with myasthenia gravis and on prescribed immunosuppressants are advised to be extra careful with the precautions of COVID‐19 such as social distancing. The risks and benefits of medications that can aggravate COVID‐19 should be weighed by the physicians and the patient together. The standard treatment options for myasthenia gravis such as pyridostigmine or 3,4‐diaminopyridine, are not proven to cause an increase in the risk of infection and therefore are recommended to be safe unless clinical findings favor otherwise. Moreover, with regards to IVIG and plasma therapy, as traveling might be required for the achievement of such therapies, careful analysis of who should get these treatments should be done. The data so far has not suggested that these agents along with monoclonal antibodies such as eculizumab are harmful with COVID‐19 infection. The risk of starting B‐cell depleting therapy might be beneficial for myasthenia gravis but not in COVID‐19 and accordingly, risks and benefits should be weighed by the physician overprescription of these drugs. A case reported that continued eculizumab use for MG after contracting COVID‐19 produced favorable outcome (Mimori M et al., 2022). As far as vaccinations are concerned, influenza and pneumococcal vaccines might help the patients and administration of only a dead vaccine is recommended in case a COVID‐19 vaccination is sought (Jacob et al., 2020). These guidelines, however, are not definitive and the publishers know, as more is being investigated about COVID‐19, these guidelines can be altered.

IVIG alone and in addition to plasmapheresis is the first‐line therapy in several autoimmune diseases such as GBS and myasthenia gravis (Lünemann et al., 2015). Accordingly, many case reports of myasthenia gravis with COVID‐19 have been documented with trials of IVIG and plasmapheresis, and have shown promising results (Aksoy & Oztutgan, 2020). According to one study, four patients were given plasma exchange or PLEX therapy and one patient received IVIG, and all of them had favorable outcomes. None of the patients who were tried for these therapies died or reported any complications, and except one patient, all were discharged without worse MGFA scores compared to their admission scores (Camelo‐Filho et al., 2020).

Another class of drugs, monoclonal antibodies, for example, eculizumab and tocilizumab, are used in the treatment of refractory myasthenia gravis and can also be used in cytokine storm crises that lead to acute respiratory distress syndrome (ARDS) related to COVID‐19. They have been used experimentally in many COVID‐19 patients and are effective in patients who had either COVID‐19‐related severe pneumonia or ARDS (Aksoy & Oztutgan, 2020). According to one study, multiple sclerosis patients showed a favorable outcome after a COVID‐19 infection while using a B‐cell depleting drug, ocrelizumab (Camelo‐Filho et al., 2020). Therefore, these drugs show promising results in myasthenia gravis and COVID‐19 patients.

Other than myasthenia gravis, the guidelines to treat other neurological manifestations of COVID‐19 are also important to discuss. A guideline has been issued by the German Neurological Society in October 2020, which mentions a wide range of management options specific to the commonly seen neurological complications in COVID‐19 infection. The guidelines pool severe studies and have 90–100% approval rates. Table 1 shows the respective treatment options most suitable for the symptoms (Berlit et al., 2020). Symptoms such as anosmia and loss of taste have limited evidence‐based treatment options and mostly resolve on their own. Benzodiazepines can be used for agitation, however, they may worsen COVID‐related respiratory complications. Delirium caused by COVID‐19 should not be treated any differently than managed in normal circumstances (Orsucci et al., 2020). In addition to this, telemedicine has been reported to be particularly helpful for (a) detection of early symptoms and signs of disease worsening, (b) prevention of life‐threatening complications, and (c) detecting early changes in the clinical picture that requires to be evaluated in a hospital setting demonstrating potential to improve patients’ management with MG during COVID‐19 pandemic (Ricciardi et al., 2021). A study also validated a Myasthenia Gravis TeleScore (MGTS), a scale for the evaluation of MG patients in telemedicine (Pasqualin et al., 2022)

**TABLE 1:** Recommendations for the management of neurological symptoms of COVID‐19

Symptom	Recommendation of treatment/management option(s)
Encephalopathy	No current evidence for specific treatment.
Meningoencephalitis	High doses of corticosteroids may be started in case of persistent symptoms.
Risk of COVID‐19 under the use of immunotherapies	Preferably, the treatment should be continued. Individual patient characteristics about the risk should be analyzed and, if needed, a change of therapy can be offered. In light of the underlying neurological illnesses, the therapy can be stopped if required.
Guillain–Barre syndrome (GBS)	CSF diagnosis is an integral part. In addition, serological testing of ganglioside antibodies should be carried out. IVIG with plasmapheresis should be started.
Acute disseminated encephalomyelitis (ADE)	New‐onset multifocal neurological symptoms suggest ADE. MRI with a contrast agent is important to recognize inflammatory lesions. A hemorrhagic complement study can be helpful. A normal CSF finding does not exclude the diagnosis.
Stroke	Detection of the possible cerebrovascular complications and prompt initiation of the necessary investigations are important. The treatment should follow the same diagnostics and treatment options as all stroke patients in acute presentation as long as hygienic measures are not compromised.
Epilepsy	An EEG should be performed and treatment of seizures carried out according to respective guidelines. However, any medication with interactions and contraindications to COVID‐19 infection should be on the look‐out. If seizures are associated with fever an NSAID should be given.
Chemosensory disturbances	They are usually expected to resolve, however if anosmia lasts for more than 3–4 weeks, an ENT and neurological assessment must be sought.
Nerve and muscle affections	Therapies to treat inflammatory diseases of musculature, the neuromuscular junction, or PNS are advised to be followed from the current guidelines. This includes the usage of plasmapheresis and IVIG. Treatment with pyridostigmine and 3,4‐diaminopyridine/ampiridine and immunomodulatory therapy (eculizumab) may be continued with special attention put to individual benefit–risk profile. Rituximab or the initiation of oral long‐term immunosuppression should be delayed depending on the clinical condition of the patient and the patient's medical history. Influenza and pneumococcal vaccination are advised also for neuromuscular patients.
Neurological intensive care medicine	Symptoms can be masked by other complications in an ICU patient and hence a thorough examination for the specific underlying neurological complications should be performed. Invasive ventilation with PEEP might be necessary regardless of increased intracranial pressure.

Abbreviations: CSF, cerebrovascular fluid; EEG, electroencephalogram; ENT, ears, nose, and throat; ICU, intensive care unit; IVIG, intravenous immunoglobulin; MRI, magnetic resonance imaging; NSAID, nonsteroidal anti‐inflammatory drug; PEEP, positive end‐expiratory pressure.

## AUTHOR CONTRIBUTIONS

All authors contributed to the study conception and design. All authors contributed to material preparation, data collection and analysis. The first draft of the manuscript was written by Syed Muhammad Ismail Shah. All authors reviewed and contributed to the intellectual content of the manuscript. All authors read and approved the final manuscript.

## CONFLICT OF INTEREST

The authors declare no conflict of interest.

### PEER REVIEW

The peer review history for this article is available at https://publons.com/publon/10.1002/brb3.2789

## Data Availability

Data are available upon reasonable request made to the first author Syed Muhammad Ismail Shah (ismailshah6551@gmail.com).

## References

[brb32789-bib-0001] Agrawal, U. , Raju, R. , & Udwadia, Z. F. (2020). Favipiravir: A new and emerging antiviral option in COVID‐19. Armed Forces Medical Journal India, 76, 370–376. 10.1016/j.mjafi.2020.08.004 PMC7467067 32895599

[brb32789-bib-0002] Aksoy, E. , & Oztutgan, T. (2020). COVID‐19 presentation in association with myasthenia gravis: A case report and review of the literature. Case Reports in Infectious Diseases, 2020, 8845844. 10.1155/2020/8845844 32850160 PMC7436352

[brb32789-bib-0003] Arumugham, V. (2019). Vaccination with bovine, chick, yeast antigens synthesizes cross‐reactive antibodies targeting human acetylcholine receptor and MuSK protein to cause myasthenia gravis: Confirmed by natural experiment (VAERS data), bioinformatics, case reports, animal experiments and titer study. 10.5281/zenodo.3421559

[brb32789-bib-0004] Assini, A. , Gandoglia, I. , Damato, V. , Rikani, K. , Evoli, A. , & Del Sette, M. (2021). Myasthenia gravis associated with anti‐MuSK antibodies developed after SARS‐CoV‐2 infection. European Journal of Neurology, 28(10), 3537–3539. 10.1111/ene.14721 33421278 PMC8014563

[brb32789-bib-0005] Auyeung, T. , Lee, J. , Lai, W. , Choi, C. H. , Lee, H. K. , Lee, J. S. , Li, P. C. , Lok, K. H. , Ng, Y. Y. , Wong, W. M. , & Yeung, Y. M. (2005). The use of corticosteroid as treatment in SARS was associated with adverse outcomes: A retrospective cohort study. Journal of Infection, 51, 98–102. 10.1016/j.jinf.2004.09.008 16038758 PMC7132384

[brb32789-bib-0006] Beigel, J. H. , Tomashek, K. M. , Dodd, L. E. , Mehta, A. K. , Zingman, B. S. , Kalil, A. C. , Hohmann, E. , Chu, H. Y. , Luetkemeyer, A. , Kline, S. , Lopez de Castilla, D. , Finberg, R. W. , Dierberg, K. , Tapson, V. , Hsieh, L. , Patterson, T. F. , Paredes, R. , Sweeney, D. A. , Short, W. R. , Lane, H. C , & ACTT‐1 Study Group Members . (2020). Remdesivir for the treatment of covid‐19—Final report. New England Journal of Medicine, 383, 1813–1826. 10.1056/NEJMoa2007764 32445440 PMC7262788

[brb32789-bib-0007] Berlit, P. , Bösel, J. , Gahn, G. , Isenmann, S. , Meuth, S. G. , Nolte, C. H. , Pawlitzki, M. , Rosenow, F. , Schoser, B. , Thomalla, G. , & Hummel, T. (2020). Neurological manifestations of COVID‐19” ‐ guideline of the German society of neurology. Neurological Research and Practice, 2, 51. 10.1186/s42466-020-00097-7 33283160 PMC7708894

[brb32789-bib-0008] Blum, S. , Lee, D. , Gillis, D. , McEniery, D. F. , Reddel, S. , & McCombe, P. (2015). Clinical features and impact of myasthenia gravis disease in Australian patients. Journal of Clinical Neuroscience, 22, 1164–1169. 10.1016/j.jocn.2015.01.022 26021730

[brb32789-bib-0009] Brooks, M. (2020). Covid‐19 linked to development of myasthenia gravis. *Medscape*. http://www.medscape.com/viewarticle/935985

[brb32789-bib-0010] Buijsers, B. , Yanginlar, C. , Maciej‐Hulme, M. L. , de Mast, Q. , & van der Vlag, J. (2020). Beneficial non‐anticoagulant mechanisms underlying heparin treatment of COVID‐19 patients. EBioMedicine, 59, 102969. 10.1016/j.ebiom.2020.102969 32853989 PMC7445140

[brb32789-bib-0011] Burrage, D. R. , Koushesh, S. , & Sofat, N. (2020). Immunomodulatory drugs in the management of SARS‐CoV‐2. Frontiers in Immunology, 11, 1844. 10.3389/fimmu.2020.01844 32903555 PMC7438578

[brb32789-bib-0012] Camelo‐Filho, A. E. , Silva, A. M. S. , Estephan, E. P. , Zambon, A. A. , Mendonça, R. H. , Souza, P. V. S. , Pinto, W. , Oliveira, A. S. B. , Dangoni‐Filho, I. , Pouza, A. F. P. , Valerio, B. C. O. , & Zanoteli, E. (2020). Myasthenia gravis and COVID‐19: Clinical characteristics and outcomes. Frontiers in Neurology, 11, 1053. 10.3389/fneur.2020.01053 33013676 PMC7516054

[brb32789-bib-0013] Chavez, A. , & Pougnier, C. (2021). A case of COVID‐19 vaccine associated new diagnosis myasthenia gravis. Journal of Primary Care & Community Health, 12, 21501327211051933. 10.1177/21501327211051933 PMC8559213 34709075

[brb32789-bib-0014] Clancy, C. J. , Buehrle, D. J. , & Nguyen, M. H. (2020). PRO: The COVID‐19 pandemic will result in increased antimicrobial resistance rates. JAC Antimicrobial Resistance, 2, dlaa049. 10.1093/jacamr/dlaa049 34192248 PMC7454644

[brb32789-bib-0015] Copaescu, A. , Smibert, O. , Gibson, A. , Phillips, E. J. , & Trubiano, J. A. (2020). The role of IL‐6 and other mediators in the cytokine storm associated with SARS‐CoV‐2 infection. Journal of Allergy and Clinical Immunology, 146, 518–534.e1. 10.1016/j.jaci.2020.07.001 32896310 PMC7471766

[brb32789-bib-0016] Delly, F. , Syed, M. J. , Lisak, R. P. , & Zutshi, D. (2020). Myasthenic crisis in COVID‐19. Journal of the Neurological Sciences, 414, 116888. 10.1016/j.jns.2020.116888 32413767 PMC7200364

[brb32789-bib-0017] De Virgiliis, F. , & Di Giovanni, S. (2020). Lung innervation in the eye of a cytokine storm: Neuroimmune interactions and COVID‐19. Nature Reviews Neurology, 16, 645–652. 10.1038/s41582-020-0402-y 32843733 PMC7446605

[brb32789-bib-0018] Evoli, A. , Alboini, P. E. , Damato, V. , Iorio, R. , Provenzano, C. , Bartoccioni, E. , & Marino, M. (2018). Myasthenia gravis with antibodies to MuSK: An update. Annals of the New York Academy of Sciences, 1412(1), 82–89. 10.1111/nyas.13518 29266255

[brb32789-bib-0019] Farina, A. , Falso, S. , Cornacchini, S. , Spagni, G. , Monte, G. , Mariottini, A. , Massacesi, L. , Barilaro, A. , Evoli, A. , & Damato, V. (2022). Safety and tolerability of SARS‐Cov‐2 vaccination in patients with myasthenia gravis: A multicenter experience. European Journal of Neurology, 29(8), 2505–2510. 10.1111/ene.15348 35390184 PMC9111609

[brb32789-bib-0020] George, J. (2020). First COVID‐19, then myasthenia gravis: Coincidence or causal? https://www.medpagetoday.com/infectiousdisease/covid19/88016

[brb32789-bib-0021] Gilhus, N. E. , Romi, F. , Hong, Y. , & Skeie, G. O. (2018). Myasthenia gravis and infectious disease. Journal of Neurology, 265, 1251–1258. 10.1007/s00415-018-8751-9 29372387

[brb32789-bib-0022] Gui, M. , Luo, X. , Lin, J. , Li, Y. , Zhang, M. , Zhang, X. , Yang, M. , Wang, W. , & Bu, B. (2015). Long‐term outcome of 424 childhood‐onset myasthenia gravis patients. Journal of Neurology, 262, 823–830. 10.1007/s00415-015-7638-2 25588729

[brb32789-bib-0023] Guidon, A. C. , & Amato, A. A. (2020). COVID‐19 and neuromuscular disorders. Neurology, 94, 959–969. 10.1212/WNL.0000000000009566 32284362

[brb32789-bib-0024] Gummi, R. R. , Kukulka, N. A. , Deroche, C. B. , & Govindarajan, R. (2019). Factors associated with acute exacerbations of myasthenia gravis. Muscle & Nerve, 60, 693–699. 10.1002/mus.26689 31469909

[brb32789-bib-0025] Hossen, M. S. , Barek, M. A. , Jahan, N. , & Islam, M. S. (2020). A review on current repurposing drugs for the treatment of COVID‐19: Reality and challenges. SN Comprehensive Clinical Medicine, 2, 1777–1789. 10.1007/s42399-020-00485-9 32904710 PMC7457893

[brb32789-bib-0026] Hübers, A. , Lascano, A. M. , & Lalive, P. H. (2020). Management of patients with generalised myasthenia gravis and COVID‐19: Four case reports. Journal of Neurology, Neurosurgery, and Psychiatry, 91, 1124–1125. 10.1136/jnnp-2020-323565 32651248

[brb32789-bib-0027] Jacob, S. , Muppidi, S. , Guidon, A. , Guptill, J. , Hehir, M. , Howard Jr, J. F. , Illa, I. , Mantegazza, R. , Murai, H. , Utsugisawa, K. , Vissing, J. , Wiendl, H. , & Nowak, R. J. (2020). Guidance for the management of myasthenia gravis (MG) and Lambert‐Eaton myasthenic syndrome (LEMS) during the COVID‐19 pandemic. Journal of the Neurological Sciences, 412, 116803. 10.1016/j.jns.2020.116803 32247193 PMC7105910

[brb32789-bib-0028] Jallouli, M. , Saadoun, D. , Eymard, B. , Leroux, G. , Haroche, J. , Huong, D. L. H. , De Gennes, C. , Chapelon, C. , Benveniste, O. , Wechsler, B. , Cacoub, P. , Amoura, Z. , Piette, J. C. , & Costedoat‐Chalumeau, N. (2012). The association of systemic lupus erythematosus and myasthenia gravis: A series of 17 cases, with a special focus on hydroxychloroquine use and a review of the literature. Journal of Neurology, 259, 1290–1297. 10.1007/s00415-011-6335-z 22160434

[brb32789-bib-0029] Jonsson, D. I. , Pirskanen, R. , & Piehl, F. (2017). Beneficial effect of tocilizumab in myasthenia gravis refractory to rituximab. Neuromuscular Disorders, 27, 565–568. 10.1016/j.nmd.2017.03.007 28433474

[brb32789-bib-0030] Kang, M. C. , Park, K. A. , Min, J. H. , & Oh, S. Y. (2022). Myasthenia gravis with ocular symptoms following a ChAdOx1 nCoV‐19 vaccination: A case report. American Journal of Ophthalmology Case Reports, 27, 101620. 10.1016/j.ajoc.2022.101620 PMID: 35800401; PMCID: PMC9254405 35800401 PMC9254405

[brb32789-bib-0031] Kusnar, L. L. , & Kaminski, H. J. (2015). Myasthenia gravis. In M. J. Zigmond , J. T. Coyle , & L. P. Rowland (Eds.), Neurobiology of brain disorders (pp. 135–150). Academic Press. 10.1016/B978-0-12-398270-4.00010-0

[brb32789-bib-0032] Lechien, J. R. , Chiesa‐Estomba, C. M. , De Siati, D. R. , Horoi, M. , Le Bon, S. D. , Rodriguez, A. , Dequanter, D. , Blecic, S. , El Afia, F. , Distinguin, L. , Chekkoury‐Idrissi, Y. , Hans, S. , Delgado, I. L. , Calvo‐Henriquez, C. , Lavigne, P. , Falanga, C. , Barillari, M. R. , Cammaroto, G. , Khalife, M. , … Saussez, S. (2020). Olfactory and gustatory dysfunctions as a clinical presentation of mild‐to‐moderate forms of the coronavirus disease (COVID‐19): A multicenter European study. European Archives of Oto‐Rhino‐Laryngology, 277, 2251–2261. 10.1007/s00405-020-05965-1 32253535 PMC7134551

[brb32789-bib-0033] Lee, M. A. , Lee, C. , Park, J. H. , & Lee, J. H. (2022). Early‐onset myasthenia gravis following COVID‐19 vaccination. Journal of Korean Medical Science, 37(10), e50. 10.3346/jkms.2022.37.e50 PMID: 35289135; PMCID: PMC8921216 35289135 PMC8921216

[brb32789-bib-0034] Li, Y. , Bai, W. , & Hashikawa, T. (2020). The neuroinvasive potential of SARS‐CoV2 may play a role in the respiratory failure of COVID‐19 patients. Journal of Medical Virology, 92, 552–555. 10.1002/jmv.25728 32104915 PMC7228394

[brb32789-bib-0035] Lünemann, J. D. , Nimmerjahn, F. , & Dalakas, M. C. (2015). Intravenous immunoglobulin in neurology–mode of action and clinical efficacy. Nature Reviews Neurology, 11, 80–89. 10.1038/nrneurol.2014.253 25561275

[brb32789-bib-0036] Luo, P. , Liu, Y. , Qiu, L. , Liu, X. , Liu, D. , & Li, J. (2020). Tocilizumab treatment in COVID‐19: A single center experience. Journal of Medicine, 92, 814–818. 10.1002/jmv.25801 PMC7262125 32253759

[brb32789-bib-0037] Lupica, A. , Di Stefano, V. , Iacono, S. , Pignolo, A. , Quartana, M. , Gagliardo, A. , Fierro, B. , & Brighina, F. (2022). Impact of COVID‐19 in AChR myasthenia gravis and the safety of vaccines: Data from an Italian cohort. Neurology International, 14(2), 406–416. https://www.ncbi.nlm.nih.gov/pmc/articles/PMC9149833/#__ffn_sectitle 35645352 10.3390/neurolint14020033 PMC9149833

[brb32789-bib-0038] Maher, D. I. , Hogarty, D. , & Ben Artsi, E. (2022). Acute onset ocular myasthenia gravis after vaccination with the Oxford‐astrazeneca COVID‐19 vaccine. Orbit, 1–5. 10.1080/01676830.2022.2062777 35499172

[brb32789-bib-0039] Melamed, R. D. , Khiabanian, H. , & Rabadan, R. (2014). Data‐driven discovery of seasonally linked diseases from an electronic health records system. BMC Bioinformatics, 15(Suppl 6), S3. 10.1186/1471-2105-15-S6-S3 PMC4158606 25078762

[brb32789-bib-0040] Mimori, M. , Komatsu, T. , Maku, T. , Mitsumura, H. , & Iguchi, Y. (2022). Generalized myasthenia gravis patients infected with COVID‐19 should continue eculizumab. Neurological Sciences, 43(7), 4081–4083. 10.1007/s10072-022-05922-2 35364770 PMC8975705

[brb32789-bib-0041] Muhammed, L. , Baheerathan, A. , Cao, M. , Leite, M. I. , & Viegas, S. (2021). MuSK antibody‐associated myasthenia gravis with SARS‐CoV‐2 infection: A case report. Annals of Internal Medicine, 174(6), 872–873. 10.7326/L20-1298 33428437 PMC7808439

[brb32789-bib-0042] Münz, C. , Lünemann, J. D. , Getts, M. T. , & Miller, S. D (2009). Antiviral immune responses: Triggers of or triggered by autoimmunity? Nature Reviews Immunology, 9, 246–258. 10.1038/nri2527 PMC2854652 19319143

[brb32789-bib-0043] Netland, J. , Meyerholz, D. K. , Moore, S. , Cassell, M. , & Perlman, S. (2008). Severe acute respiratory syndrome coronavirus infection causes neuronal death in the absence of encephalitis in mice transgenic for human ACE2. Journal of Virology, 82, 7264–7275. 10.1128/JVI.00737-08 18495771 PMC2493326

[brb32789-bib-0044] Neykova, K. K. , Milanova, M. , & Ignatov, P. N. (2021). Myasthenia gravis and covid‐19 in pregnancy: A review of the literature and case series report. The Journal of Maternal‐Fetal & Neonatal Medicine, 1–9. 10.1080/14767058.2021.1973418 34582289

[brb32789-bib-0045] NIH . (2020). Hydroxychloroquine does not benefit adults hospitalized with COVID‐19. National Institutes of Health. https://www.nih.gov/news‐events/news‐releases/hydroxychloroquine‐does‐not‐benefit‐adults‐hospitalized‐covid‐19

[brb32789-bib-0046] Orsucci, D. , Ienco, E. C. , Nocita, G. , Napolitano, A. , & Vista, M. (2020). Neurological features of COVID‐19 and their treatment: A review. Drugs in Context, 9, 1–12. 10.7573/dic.2020-5-1 PMC7295105 32587625

[brb32789-bib-0047] Pasqualin, F. , Guidoni, S. V. , Albertini, E. , Ermani, M. , Frangiamore, R. , Vanoli, F. , Antozzi, C. , Mantegazza, R. , & Bonifati, D. M. (2022). Development and validation of the Myasthenia Gravis Telescore (MGTS). Neurological Sciences, 43(7), 4503–4509. 10.1007/s10072-022-05918-y 35226211 PMC8883006

[brb32789-bib-0048] Phillips, W. D. , & Vincent, A. (2016). Pathogenesis of myasthenia gravis: Update on disease types, models, and mechanisms. F1000Res, 5, 1513. 10.12688/f1000research.8206.1 PMC4926737 27408701

[brb32789-bib-0049] Pradhan, S. , Pardasani, V. , & Ramteke, K. (2009). Azithromycin‐induced myasthenic crisis: Reversibility with calcium gluconate. Neurology India, 57, 352–353. 10.4103/0028-3886.53270 19587486

[brb32789-bib-0050] Ramos‐Fransi, A. , Rojas‐García, R. , Segovia, S. , Márquez‐Infante, C. , Pardo, J. , Coll‐Cantí, J. , Jericó, I. , Illa, I. , & Myasthenia NMD‐ES Study Group . (2015). Myasthenia gravis: Descriptive analysis of life‐threatening events in a recent nationwide registry. European Journal of Neurology, 22, 1056–1061. 10.1111/ene.12703 25847221

[brb32789-bib-0051] Rein, N. , Haham, N. , Orenbuch‐Harroch, E. , Romain, M. , Argov, Z. , Vaknin‐Dembinsky, A. , & Gotkine, M. (2020). Description of 3 patients with myasthenia gravis and COVID‐19. Journal of the Neurological Sciences, 417, 117053. 10.1016/j.jns.2020.117053 32731059 PMC7832365

[brb32789-bib-0052] Restivo, D. A. , Centonze, D. , Alesina, A. , & Marchese‐Ragona, R. (2020). Myasthenia gravis associated with SARS‐CoV‐2 infection. Annals of Internal Medicine, 173, 1027–1028. 10.7326/L20-0845 32776781 PMC7429993

[brb32789-bib-0053] Ricciardi, D. , Casagrande, S. , Iodice, F. , Orlando, B. , Trojsi, F. , Cirillo, G. , Clerico, M. , Bozzali, M. , Leocani, L. , Abbadessa, G. , Miele, G. , Tedeschi, G. , Bonavita, S. , Lavorgna, L. , & Digital Technologies, Web, Social Media Study Group of the Italian Society of Neurology . (2021). Myasthenia gravis and telemedicine: A lesson from COVID‐19 pandemic. Neurological Sciences, 42(12), 4889–4892. 10.1007/s10072-021-05566-8 34436726 PMC8390022

[brb32789-bib-0054] Rizk, J. G. , Kalantar‐Zadeh, K. , Mehra, M. R. , Lavie, C. J. , Rizk, Y. , & Forthal, D. N. (2020). Pharmaco‐immunomodulatory therapy in COVID‐19. Drugs, 80, 1267–1292. 10.1007/s40265-020-01367-z 32696108 PMC7372203

[brb32789-bib-0055] Ruan, Z. , Tang, Y. , Li, C. , Sun, C. , Zhu, Y. , Li, Z. , & Chang, T. (2021). COVID‐19 vaccination in patients with myasthenia gravis: A single‐center case series. Vaccines, 9(10), 1112. 10.3390/vaccines9101112 34696220 PMC8537454

[brb32789-bib-0056] Sanders, J. M. , Monogue, M. L. , Jodlowski, T. Z. , & Cutrell, J. B. (2020). Pharmacologic treatments for coronavirus disease 2019 (COVID‐19). JAMA, 323, 1824–1836. 10.1001/jama.2020.6019 32282022

[brb32789-bib-0069] Saied, Z. , Rachdi, A. , Thamlaoui, S. , Nabli, F. , Jeridi, C. , Baffoun, N. , Kaddour, C. , Belal, S. , & Ben Sassi, S. (2021). Myasthenia gravis and COVID‐19: A case series and comparison with literature. Acta Neurologica Scandinavica, 144(3), 334–340. Portico. 10.1111/ane.13440 33914898 PMC8222886

[brb32789-bib-0057] Scavone, C. , Brusco, S. , Bertini, M. , Sportiello, L. , Rafaniello, C. , Zoccoli, A. , Berrino, L. , Racagni, G. , Rossi, F. , & Capuano, A. (2020). Current pharmacological treatments for COVID‐19: What's next? British Journal of Pharmacology, 177, 4813. 10.1111/bph.15072 32329520 PMC7264618

[brb32789-bib-0058] Singhal, T. (2020). A review of coronavirus disease‐2019 (COVID‐19). Indian Journal of Pediatrics, 87, 281–286. 10.1007/s12098-020-03263-6 32166607 PMC7090728

[brb32789-bib-0059] Solé, G. , Salort‐Campana, E. , Pereon, Y. , Stojkovic, T. , Wahbi, K. , Cintas, P. , Adams, D. , Laforet, P. , Tiffreau, V. , Desguerre, I. , Pisella, L. I. , Molon, A. , Attarian, S. , & FILNEMUS COVID‐19 Study Group . (2020). Guidance for the care of neuromuscular patients during the COVID‐19 pandemic outbreak from the French Rare Health Care for Neuromuscular Diseases Network. Revista De Neurologia, 176, 507–515. 10.1016/j.neurol.2020.04.004 PMC7167585 32354651

[brb32789-bib-0068] Taheri, A. , Davoodi, L. , Soleymani, E. , & Ahmadi, N. (2022). New‐onset myasthenia gravis after novel coronavirus 2019 infection. Respirology Case Reports, 10(6). Portico. 10.1002/rcr2.978 PMC9125167 35620352

[brb32789-bib-0060] Toscano, G. , Palmerini, F. , Ravaglia, S. , Ruiz, L. , Invernizzi, P. , Cuzzoni, M. G. , Franciotta, D. , Baldanti, F. , Daturi, R. , Postorino, P. , Cavallini, A. , & Micieli, G. (2020). Guillain–Barré syndrome associated with SARS‐CoV‐2. New England Journal of Medicine, 382, 2574–2576. 10.1056/NEJMc2009191 32302082 PMC7182017

[brb32789-bib-0061] Tugasworo, D. , Kurnianto, A. , Retnaningsih , Andhitara, Y. , Ardhini, R. , & Budiman, J. (2022). The relationship between myasthenia gravis and COVID‐19: A systematic review. Egypt Journal of Neurology, Psychiatry and Neurosurgery, 58, 83. 10.1186/s41983-022-00516-3 PMC9261189 35818475

[brb32789-bib-0062] Varan, O. , Kucuk, H. , & Tufan, A. (2015). Myasthenia gravis due to hydroxychloroquine. Reumatismo, 67, 849. 10.4081/reumatismo.2015.849 26876193

[brb32789-bib-0063] Varatharaj, A. , Thomas, N. , Ellul, M. A. , Davies, N. W. S. , Pollak, T. A. , Tenorio, E. L. , Sultan, M. , Easton, A. , Breen, G. , Zandi, M. , Coles, J. P. , Manji, H. , Al‐Shahi Salman, R. , Menon, D. K. , Nicholson, T. R. , Benjamin, L. A. , Carson, A. , Smith, C. , Turner, M. R. , … Michael, B. D , & CoroNerve Study Group . (2020). Neurological and neuropsychiatric complications of COVID‐19 in 153 patients: A UK‐wide surveillance study. Lancet Psychiatry, 7, 875–882. 10.1016/S2215-0366(20)30287-X 32593341 PMC7316461

[brb32789-bib-0064] Wendell, L. C. , & Levine, J. M. (2011). Myasthenic crisis. Neurohospitalist, 1, 16–22. 10.1177/1941875210382918 23983833 PMC3726100

[brb32789-bib-0065] Xu, X. , Han, M. , Li, T. , Sun, W. , Wang, D. , Fu, B. , Zhou, Y. , Zheng, X. , Yang, Y. , Li, X. , Zhang, X. , Pan, A. , & Wei, H. (2020). Effective treatment of severe COVID‐19 patients with tocilizumab. PNAS, 117, 10970–10975. 10.1073/pnas.2005615117 32350134 PMC7245089

[brb32789-bib-0066] Zhao, H. , Shen, D. , Zhou, H. , Liu, J. , & Chen, S. (2020). Guillain–Barré syndrome associated with SARS‐CoV‐2 infection: Causality or coincidence? The Lancet Neurology, 19, 383–384. 10.1016/S1474-4422(20)30109-5 32246917 PMC7176927

